# The Work-Family Spillover Effects of Customer Mistreatment for Service Employees: The Moderating Roles of Psychological Detachment and Leader–Member Exchange

**DOI:** 10.3389/fpsyg.2019.02107

**Published:** 2019-09-18

**Authors:** Ran Zhang, Yunqiao Wu, Karen Ferreira-Meyers

**Affiliations:** ^1^Faculty of Economics and Management, East China Normal University, Shanghai, China; ^2^School of Business and Management, Shanghai International Studies University, Shanghai, China; ^3^Institute of Distance Education, University of Eswatini, Kwaluseni, Swaziland

**Keywords:** customer mistreatment, work-to-family conflict, psychological detachment, leader–member exchange, family satisfaction

## Abstract

Past literature in the area of employee–customer interactions suggests that being mistreated by customers is deemed one of the most important work-related stressors for service employees. However, little is known about the effects of customer mistreatment on the family domain. In a representative sample of 221 front-line employees in the East China hairdressing industry using three separate surveys administered 1 month apart respectively, the current study explores the mediation effects of work-to-family conflict (WFC) and the moderation effects of psychological detachment (PD) and leader–member exchange (LMX) on the relationship between customer mistreatment and family satisfaction (FS). The research revealed that the employees confronted with intensive customer mistreatment tended to experience high levels of WFC, and WFC mediated the effects of customer mistreatment on FS. In addition, both PD and LMX attenuated customer mistreatment’s direct effects on WFC and indirect effects on FS (via WFC). This study contributes to the managerial psychology literature related to the customer mistreatment construct and a better understanding of how PD and LMX act as a work-family spillover effect moderator of customer mistreatment on individuals.

## Introduction

Scholarly work has identified that customer mistreatment was a daily occurrence in the service industry (e.g., Grandey et al., 2004, 2007), and likely represented the most important future source of work stress for service workers (Dudenhöffer and Dormann, 2013). Customer mistreatment refers to the low-quality interpersonal treatment that service workers receive from customers (Wang et al., 2011), and it comes in many forms, including verbal bullying, disrespectful behavior, or unjust demands of customers that one employee serves (Chi et al., 2018). For service workers, being “nice” to uncivil customers is mandated behavior (Rupp et al., 2008), whilst customers, due to their higher relative power, may feel justified in mistreating employees rather than feeling required to reciprocate such friendliness. With the mantra “the customer is always right,” the mistreatment by customers is further intensified (Yagil, 2008).

In the past decade, emerging studies have demonstrated that customer mistreatment can have negative consequences in terms of victim well-being (e.g., Grandey et al., 2004; Chi et al., 2018; Park and Kim, 2019) and work behavior (e.g., Skarlicki et al., 2008; Shao and Skarlicki, 2014; Baranik et al., 2017; Garcia et al., 2019). Despite these fruitful findings, the effects of customer mistreatment on the family domain for service employees remain almost unexplored, and this lack of knowledge is problematic, for family is one of the most important non-work domain which strongly relates to one’s well-being, work attitude and behavior (Casper et al., 2007). Zhang et al. (2016) also call for studies to illustrate how customer-related stressors adversely affect the targets’ family domain.

To address this gap, the purpose of this study is thus twofold. On the one hand, we examine the process underlying the linkage between customer mistreatment and family satisfaction. Family satisfaction refers to the extent to which one is satisfied with one’s family life or situation (Rathi and Barath, 2013) and is viewed as a central indicator of individual well-being (Page and Vella-Brodrick, 2009). According to the work-family interface model proposed by Liu et al. (2013), work-to-family conflict (WFC) has been identified as an important mediator that links job stressors and family outcome (Aryee et al., 1999; Ford et al., 2007), and the current study thus specifically addressed WFC’s mediation in the customer mistreatment-family satisfaction relationship. In addition, we used the analytic framework of stressor-strain-outcome (SSO, Koeske and Koeske, 1993) in the study to investigate the relations between customer mistreatment, WFC, and family satisfaction. According to this analytic framework, stressors lead to strain, and strain impacts on outcomes. Thus, by applying the SSO model, we posit that, for service employees, customer mistreatment (i.e., stressor) is associated with WFC (i.e., strain), which, in turn, eventually relates to family satisfaction (i.e., outcome).

On the other hand, in response to Chi et al.’s (2018) call to explore how to attenuate customer mistreatment’s harmful consequences, we examine two moderating variables – one individual trait variable (i.e., psychological detachment) and one social resource variable (i.e., leader-member exchange, LMX) – in buffering the customer mistreatment’s work-family spillover effects for service employees. Psychological detachment is defined as an “‘individual’ sense of being away from the work situation” (Etzion et al., 1998, p. 579), and can facilitate recovery from job stressors (Sonnentag and Bayer, 2005). Broadly deemed a form of adaptive strategy to cope with job stressors (e.g., Sonnentag et al., 2008a), psychological detachment may attenuate the impacts of customer mistreatment on individuals. Leader–member exchange refers to quality relationships between supervisors and subordinates, and is viewed as a social resource able to minimize negative employment experiences (O’Driscoll et al., 2003; Erdogan et al., 2004). Surprisingly, until now, the manner in which psychological detachment or LMX moderates customer mistreatment effects has not been addressed in the academic field. In this study we expect that employees who can be psychologically detached during off-job time or those with high LMX are less prone to react to customer mistreatment by experiencing WFC, and thereby likely to experience satisfaction in their family life.

## Theoretical Background and Hypotheses

### The Customer Mistreatment-WFC Relationship

In Koeske and Koeske’s (1993) SSO framework, stressors are environmental stimuli (e.g., work events) and perceived as troublesome and challenging for service employees, while strains are negative reactions tied to situational stimuli (i.e., the reputed results of stressor exposure). Customer mistreatment is a significant job stressor for many service jobs (Dormann and Zapf, 2004; Chi et al., 2018). However, little research has focused on this service job stressor in relation to the strains in the family domain (e.g., WFC). Based on the stressor–strain relationship from the SSO framework, the current study hypothesizes that customer mistreatment (stressor) positively relates to WFC (strain). Work-family conflict is “a form of inter-role conflict in which the role pressures from the work and family domains are mutually incompatible in some respects” (Greenhaus and Beutell, 1985, p. 77), and WFC occurs when demands and situations in the work domain interfere with the family domain. As a kind of family related strain, work-family conflict represents one’ aversive reaction to the stresses he or she experiences in the workplace. In the current study, the stressor-strain relationship (i.e., customer mistreatment-WFC relationship) can be explained by Hobfoll’s (1989, 2001) Conservation of resource (COR) theory which is viewed as an important tool to explain the stressor-strain relationship (Halbesleben et al., 2014).

The COR theory (Hobfoll, 1989, 2001) posits that (a) an individual’ s resources are limited and he or she thus strives to retain, protect, and establish resources, and (b) stress will occur if there is a lack of resource gain following an investment. Employees invest their limited resources in the customer interactions for their well-being, such as self-efficacy and/or recognition of social skills (Brotheridge and Grandey, 2002; Zapf, 2002). However, serving customers may have downsides for employees. In the service process, the frequent interaction with negatively behaving customers is viewed as a stress-related construct (e.g., Grandey et al., 2004; Kim et al., 2012), and individual resources may be depleted by exposure to customer-related stressors. Past research has shown that, for service employees, the investment of the limited resources for their well-being gains poses a great risk in the confrontation with negative customers (e.g., Dormann and Zapf, 2004). For instance, employees’ self-efficacy may decrease and their optimism may diminish if customer expectations can rarely be satisfied (Dormann and Zapf, 2004). Besides, the unpleasant social interaction with misbehaving customers will give rise to negative feelings such as anger and anxiety (Song et al., 2018). However, service employees need to suppress their negative feelings to follow display rules set by organizations (Grandey et al., 2004; Garcia et al., 2019). For instance, service employees are required to engage in “service-with-a-smile” during the interaction even when being exposed to customer mistreatment. For service employees, to balance one’s own reactions to customer mistreatment to those required by organizations is a challenging task, and individual resources such as energy will be drained to cope with the negative experiences of customer mistreatment. As such, a net loss in one’s resources may arise for the individual targeted by customer mistreatment. According to the COR theory, with the attempt to conserve the limited resources, one may likely deploy strategies to prevent further loss of one’s personal resources, and one effective way is to decrease the time and energy to be dedicated to the family domain. That is, an individual experiencing intensive customer mistreatment might leave fewer resources available for family demands, engendering WFC increase. Just as Byron (2005) argued, the more time or energy one spends in a role, the more interference in the secondary role. Early on, Goode (1960) also argued that the ability of employees to successfully manage their responsibilities in the non-work domain (e.g., family domain) will be limited by work-domain demands (e.g., interacting with negatively behaving customers). In addition, from the point-view of emotions, it has been argued (Chi et al., 2018) that individuals who experience customer mistreatment in the workplace may take negative emotions resulting from this mistreatment back home, engendering work-family conflict. Park and Kim (2019) also argued that customer mistreatment’s harmful effects can be extended to the personal life domain. In summary, service employees who are mistreated by customers are prone to experience WFC. Hence, it can be expected that:

**H1** Customer mistreatment positively relates to WFC.

### The WFC- Family Satisfaction Relationship

In the SSO framework, outcomes are referred to as “enduring behavioral or psychological consequences of prolonged stress and strain” (Koeske and Koeske, 1993, p. 111), and include attitudes and behaviors. In the current study, family satisfaction is defined as an attitudinal outcome that is elicited by WFC (i.e., family related strain). In line with the strain-outcome relationship from Koeske and Koeske’s (1993) SSO framework, we proposed that WFC negatively predicts family satisfaction. Work and family roles are the two most important life roles for most people, and the incompatibility between these two roles may create tension and negative feelings (Grandey et al., 2005). Several studies have provided empirical evidence that WFC is negatively predicative of family satisfaction (e.g., Liu et al., 2013; Rathi and Barath, 2013). In the current study, the resource perspective can be applied to explain how individuals react to WFC experiences.

Family resources include a stable family life, intimacy with family, time for family and so on (Hobfoll, 2001), and people invest their resources in the family domain for one’s well-being. For instance, time shared with family members (e.g., watching TV together) can give one a sense of family life satisfaction (Rathi and Barath, 2013), and handling family-related issues well (e.g., fixing home furniture) may enhance one’s self-worth. However, WFC signifies decrease of resources allocated to the family domain. For instance, the time-based WFC conflict occurs when the time required by the family domain is occupied by work-related affairs. Thus, individuals experiencing WFC might lack the resources (e.g., time, energy, or emotion) to participate in family activities or responsibilities. Such negative social interactions with family may create a sense of threat to one’s self and a reduced sense of well-being. In addition, those hassled by WFC are prone to engage in family undermining behavior (e.g., displacing anger toward family members) (Wu et al., 2012), and these negative family related experiences may damage the self-worth and quality of family life for family members, and bring about a cold, unfriendly place without solidarity (Xin et al., 2018). It is thus clear that WFC experiences play an important role in ruining the home environment and then decrease one’s family satisfaction in the family domain. Based upon this, the following hypothesis is proposed:

**H2** WFC negatively relates to family satisfaction.

### WFC’s Mediating Role

Given that customer mistreatment may be positively associated with WFC in the stressor-strain relationship and that WFC may negatively relate to family satisfaction in the strain-outcome relationship from Koeske and Koeske’s (1993) SSO framework, WFC is thus expected to link customer mistreatment and family satisfaction. Hence, we hypothesize:

**H3** WFC mediates the relationship between customer mistreatment and family satisfaction.

### Psychological Detachment’s Moderating Role

Psychological detachment refers to the process of temporarily disconnecting oneself mentally or psychologically from work during after-work hours. For an individual, unwinding from work is an effective approach to buffer job stressors’ negative impacts (e.g., customer mistreatment) on the self (De Croon et al., 2004). Thus, to be psychologically detached from negative work experiences rather than to remain fixated on them during off-job time, may be an adaptive coping mechanism that helps one’s recovery from mistreatment-triggered fatigue and thus prevent its spill-over effects on one’s family domain.

Psychological detachment may affect employees’ reactions to customer mistreatment, and it can be explained from the resource point of view. Detachment during non-work time means that one will not think about work during non-work time, and thus job stressors (i.e., customer mistreatment) that individuals encounter in the workplace cease to impact on them during off-work time. For instance, Sonnentag and Fritz (2007) argued that being away from the negative work situations can protect one from being influenced by additional job demands. For individuals of high psychological detachment, the duration of customer mistreatment will not be prolonged beyond the work-time boundary, and then its initial negative effects on the family domain are less likely to be lengthened. As such, the further loss of one’s personal resources resulting from customer mistreatment may be prevented. Conversely, individuals who lack psychological detachment tend to ruminate on the negative job experiences for an extended period of time (e.g., the time at home), and job stressors (i.e., customer mistreatment) then continue to deplete their resources (normally intended for family life) after regular work hours, thus engendering more WFC. Besides, the positive state of mind that psychological detachment brings can conserve one’s existing internal resources such as energy and confidence (Sonnentag and Fritz, 2007). Specifically, a positive state of mind enables individuals to be engaged in happy family activities (seeing movies with family members) that might offer them resources (e.g., affective resources). Sonnentag et al. (2008b) also argued that psychological detachment from work during off-job time is an effective way to help one to build up personal resources such as energy and emotion. That is, the individuals with high levels of psychological detachment that can conserve and even supplement resources, and they thus are less likely to experience WFC. Based upon this, we hypothesize:

**H4** Psychological detachment moderates the relationship between customer mistreatment and WFC.

### Leader–Member Exchange’s Moderating Role

LMX is the construct that focuses on the dyadic relationship between supervisors and subordinates. Each supervisor is prone to develop relationships of different quality levels with subordinates, and thus subordinates are treated differently by their supervisors (Graen and Uhl-Bien, 1995). A high-quality LMX relationship relies on mutually reciprocated supervisor-subordinate social exchanges and is demonstrated by increased levels of trust, support, and mutual respect between two parties. On the contrary, in a low-quality LMX relationship, the supervisor-subordinate exchange is “contractual,” and both supervisor and subordinate show less trust, care and support to each other (Martin et al., 2015; Pan and Lin, 2018). The existing social support literature indicates that support buffers the stressor-strain relationship (Cohen and Wills, 1985; Viswesvaran et al., 1999). High-quality LMX relationships might attenuate the relationship between stressor (i.e., customer mistreatment) and strain (i.e., WFC). In the current study, the moderating role of LMX in the customer mistreatment-WFC relationship will be specifically explained from the social resource point of view.

High-quality LMX relationships have been deemed one of the most important social resources for employees (Lee and Ashforth, 1996). In a high-quality LMX relationship, supervisors intend to offer subordinates valuable resources that can be both intangible (e.g., trust, recognition and support) and tangible (e.g., protection from unfair practices, information and feedback) (Martinaityte and Sacramento, 2013). For instance, subordinates with high-quality LMX relationships are more likely to receive better job-related information and more objective performance ratings (Kacmar et al., 2003). According to the COR theory (Hobfoll, 1989), a high-quality LMX relationship can facilitate the conserving of one’s resource reservoir, and thus serves as an important resource for one to resist stress. Upon experiencing customer mistreatment, high-LMX individuals may have access to their supervisors’ strong backing for customer service behaviors (e.g., denying service to uncivil customers) and protection against unreasonable customer complaints. Tangirala et al. (2007) argued that someone with a high-quality LMX relationship can have access to supervisory support (e.g., supervisory recognition) that might partly offset his or her perceptions of not being appreciated by customers. Moreover, in a high-quality LMX relationship, the leaders intend to have more trusted and supportive communications with their followers, and these communications can assist in decreasing the followers’ feeling of loss, frustration and even anger as a result of the frequent interactions with uncivil customers, thus engendering a positive appraisal of customer service (e.g., interpreted as an opportunity of self-challenge) for the followers. Employees with high-quality LMX relationships are more prone to favorably viewing their customers (Medler-Liraz and Kark, 2012). It is thus clear that employees in high-quality LMX relationships are provided with social resources by their supervisors which can mitigate the adverse influence of customer mistreatment to them. On the contrary, low LMX employees are called “out-group” members, and they are less likely to receive the valuable supervisory resources (e.g., trust, support, communications, and feedback) for the interactions with the disliked and uncivil customers. As such, based on COR theory, compared with their counterpart, the high LMX individuals have access to more resources from their supervisors which may help to conserve and supplement the already present resources, and they are thus less likely to experience WFC. Thus, we hypothesize:

**H5** LMX moderates the relationship between customer mistreatment and WFC.

### A Moderated Mediation Framework

**FIGURE 1:**
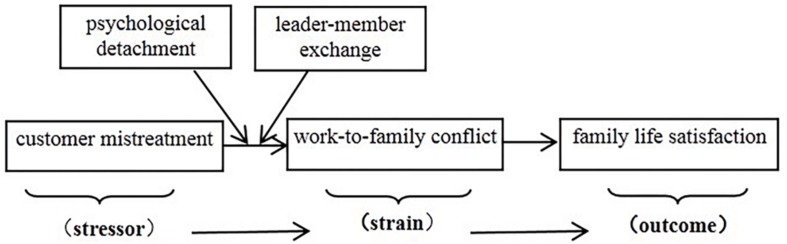
The conceptual model of the study.

The mediation role of WFC in the customer mistreatment-family satisfaction relationship and the moderating roles of psychological detachment and LMX in the customer mistreatment-WFC relationship suggest that the two aforementioned moderators will conditionally influence the strength of the indirect relationship between customer mistreatment and family satisfaction (via WFC). This reflects a pattern of moderated mediation between the current study variables, which is shown in Figure 1. We posit that WFC’s mediating effects (Hypothesis 3) will be weaker when psychological detachment or LMX is high. Thus, we hypothesize:

**H6** The indirect effect of customer mistreatment on family satisfaction through WFC is weaker when psychological detachment is higher.

**H7** The indirect effect of customer mistreatment on family satisfaction through WFC is weaker when LMX is higher.

## Materials and Methods

### Sample and Procedure

An ethics approval was not required because no unethical behaviors existed in the procedures for the current study. Nevertheless, the written informed consent was obtained from all participants, and the protocol followed the ethical guideline of the American Psychological Association as well as the recommendations by our local ethical review board.

We collected data from frontline service employees at a big beauty and hairdressing company of more than 8000 employees located in East China. The participating company helped to pull a random sample from the full-time service employees that interact directly with customers (i.e., barbers/beauticians), and assisted us in distributing the research announcement to these employees, along with an informative letter emphasizing the confidentiality and anonymity of the responses. Finally 276 voluntarily agreed to participate in the surveys. These 276 barbers/beauticians, as the boundary spanning employees in beauty and hairdressing industry, have direct and frequent interactions with the customers and therefore they are confronted with the mistreatment by customers, such as being verbally abused by customers for the dissatisfaction with hair-cutting quality, and demanding or unreasonable requests from customers.

To clarify the causal inference and alleviate the problem of common method variance (CMV), we separated measurement occasions. The data were collected at three-time points, 1 month apart. The Time 1 survey measured self-reported customer mistreatment and collected demographic information of the participants, and the Time 2 survey measured WFC, psychological detachment and leader-member exchange, and Time 3 survey measured family satisfaction. The paper-based surveys of three stages were distributed to the participants during breaking time in the workplace, and they were asked to return the completed questionnaire in a sealed envelope to the on-site researchers. No monetary incentive was offered for the participants. The employee payroll number were used to match the questionnaires of respondents. Of 276 participants, 221 validly completed surveys at all time points (80% response rate). Among these participants, the majority were male (57%), and the average age was 26.96 years (*SD* = 5.84), and 39% of the participants were married (*SD* = 0.49).

### Measurement

All scale items were in the form of a statement followed by a five-point response array ranging from 1 (strongly disagree) to 5 (strongly agree). We followed the translation back-translation procedures to translate the English-based measures into Chinese.

#### Customer Mistreatment

Customer mistreatment was measured by the 8-item scale developed by Shao and Skarlicki (2014). One sample item is “Criticized me in front of my colleagues or supervisors.” The reliability of this scale is 0.84.

#### Work-to-Family Conflict

Work-to-family conflict was measured using the 9-item scale developed by Carlson et al. (2000). A sample item is “My work keeps me from my family activities more than I would like.” The reliability of this scale is 0.96.

#### Psychological Detachment

Psychological detachment was measured by the 4-item scale developed by Sonnentag and Fritz (2007). One sample item is “During after-work hours, I forget about work.” The reliability of this scale is 0.91.

#### Leader–Member Exchange

Leader–member exchange was measure using a 7-item scale developed by Scandura and Graen (1984). A sample item is ‘My general manager will use his/her power to help me solve problems in my work.’ The reliability of this scale is 0.95.

#### Family Satisfaction

Family satisfaction was measured by the 4-item scale developed by Mills et al. (1992). One sample item is “How satisfied are you with your relationship with your family?”. The reliability of this scale is 0.82.

#### Control Variables

In the current study, the variables including age, gender, and marital status were controlled due to their potential effects to family related constructs such as WFC (Baron and Kenny, 1986; Deng and Gao, 2017).

## Results

### Descriptive Statistics and Correlations

Table 1 shows the descriptive statistics and inter-correlations of the study variables, which provided initial support for the study’s hypotheses. As expected, all bivariate correlations for variables linked through the hypotheses were statistically correlated in the anticipated direction. For instance, customer mistreatment correlated positively with WFC (*r* = 0.35, *p* < 0.01) and WFC correlated negatively with family satisfaction (*r* = −0.27, *p* < 0.01).

**TABLE 1:** Mean, SD, and Person correlation for study variables.

**Variable**	***M***	***SD***	**1**	**2**	**3**	**4**	**5**	**6**	**7**	**8**
(1) Gender	0.57	0.50	1							
(2) Age	26.96	5.84	0.01	1						
(3) Marital status	0.39	0.49	0.01	0.37^∗∗^	1					
(4) Customer mistreatment (Time 1)	2.93	0.72	–0.01	0.01	0.01	1				
(5) Psychological detachment (Time 2)	3.18	0.87	–0.05	–0.06	–0.05	0.04	1			
(6) Leader-member exchange (Time 2)	3.20	0.72	–0.03	–0.06	–0.05	0.45^∗∗^	0.46^∗∗^	1		
(7) Work-to-family conflict(Time 2)	2.84	0.81	–0.02	0.05	0.04	0.35^∗∗^	–0.41^∗∗^	–0.34^∗∗^	1	
(8) Family satisfaction(Time 3)	3.58	0.68	–0.02	0.04	0.05	−0.15^∗^	–0.16^∗∗^	−0.15^∗^	−0.27^∗^	1

**N* = 221; ^∗^*p* < 0.05; ^∗∗^*p* < 0.01; *M*, Mean; *SD*, Standard Deviation.*

### Confirmatory Factor Analysis

To ensure sufficient convergent and discriminant validity among all constructs, we created the full measurement model (i.e., the five-factor model) against a range of alternative models by conducting a series of confirmatory factor analyses (CFA). As can be seen in Table 2, the five-factor model provided a good fit to the data [χ^2^ (454) = 681.16, *p* < 0.01, *χ*^2^/df = 1.50, RMSEA = 0.05, CFI = 0.96, IFI = 0.96]. Compared with other four models, the five-factor model produced a significant improvement in chi-squares, suggesting a better fit. Besides, the five-factor model is the only model with the values for RMSEA, IFI and CFI that reach the recommended criterions (Bentler, 1990). Thus, the respondents could distinguish all the five constructs (i.e., Customer mistreatment, WFC, LMX, Psychological detachment, family satisfaction) clearly in the current study.

**TABLE 2:** Measurement model comparisons.

**Model**	***df***	***χ*^2^**	**RMSEA**	**CFI**	**IFI**	**Δ *χ*^2^**
Five-factor model	454	681.16^∗∗^	0.05	0.96	0.96	
(full measurement)						
Four-factor model^a^	458	1185.68^∗∗^	0.09	0.87	0.87	504.52
Four-factor model^b^	458	1750.48^∗∗^	0.11	0.77	0.77	1069.32
Three-factor model^c^	461	2253.74^∗∗^	0.13	0.68	0.68	1572.58
One-factor model^d^	464	3833.37^∗∗^	0.18	0.40	0.40	3152.21

**N* = 221, ^∗∗^*p* < 0.01; *χ*2, chi-square discrepancy; *df*, degrees of freedom; IFI, incremental fit index; CFI, comparative fit index; RMSEA, root mean square error of approximation. ^*a*^LMX and psychological detachment combined into a single factor. ^*b*^Customer mistreatment and WFC combined into a single factor. ^*c*^Customer mistreatment and WFC combined into a single factor; LMX and Psychological detachment combined into a single factor. ^*d*^All variables combined into a single factor.*

### Test of Hypotheses

In the current study, the hierarchical multiple regression was used to test Hypotheses 1–3, and hierarchical moderated regression to test Hypotheses 4–5. We tested the moderated mediation (Hypotheses 6–7) by the analytic path procedures (Preacher et al., 2007). Hayes’ PROCESS tool (Hayes, 2013) was used to estimate both the mediation and moderated mediation models, and bootstrapping analysis was conducted to assess the significance of indirect effects (Shrout and Bolger, 2002).

#### Testing of Direct and Mediation Effects

Table 3 presents the results for *H1* and *H2*. Supporting *H1*, customer mistreatment was significantly and positively associated with WFC (β = 0.35, *p* < 0.01, see *first stage* in Table 3). In addition, after controlling for customer mistreatment, WFC was still significantly and negatively associated with family satisfaction (β = –0.26, *p* < 0.01, see *second stage* in Table 3). Thus, *H2* was also supported.

**TABLE 3:** Regression results for main effect and mediation effect.

**Variable**	**First stage**		**Second stage**	
	**(dependent**		**(dependent**	
	**variable = WFC)**		**variable = FS)**	
		
	***Step 1***	***Step 2***	***Step 1***	***Step 2***
Gender	–0.02	–0.01	–0.03	–0.03
Age	0.04	0.04	0.03	0.04
Marital status	0.03	0.02	0.04	0.05
Customer mistreatment		0.35^∗∗^	–0.15^∗∗^	–0.06
WFC				–0.26^∗∗^
*R*^2^	0.01	0.11^∗∗^	0.03^∗∗^	0.08^∗∗^
Δ *R*^2^		0.10		0.05

**Bootstrapping results for indirect effects**		Estimate	*SE*	95% CI
Customer mistreatment →WFC→FS		−0.09	0.03	[−0.16, −0.04]
**Sobel testing results for indirect effects**		Estimate	*SE*	Z
Customer mistreatment →WFC→FS		−0.09	0.03	−3.05^∗∗^
**Bootstrapping results for direct effects**		Estimate	*SE*	95% CI
Customer mistreatment →FS, controlling for WFC		−0.06	0.07	[– 0.19, 0.08]

**N* = 221; ^∗^*p* < 0.05; ^∗∗^*p* < 0.01; WFC, work-to-family conflict; FS, family satisfaction; CI, confidence interval; LL, lower limit; UL, upper limit. Bootstrap sample size = 1000.*

As supposed by MacKinnon et al. (2007), there already exists some support for the possibility of an indirect effect, for both direct effects of customer mistreatment on WFC (*H1*) and those of WFC on family satisfaction (*H2*) were significant. Besides this, when controlling WFC, customer mistreatment was not significantly associated with family satisfaction (see *first and second stage* in Table 3). As Baron and Kenny (1986) argued, full mediation exists if the effect of the independent variable on the dependent variable becomes void (when the mediator is added). As such, the possibility of full WFC mediation between customer mistreatment and family satisfaction exists.

To further validate the significance of WFC’s mediation, we used both the Sobel test (Sobel, 1982) and bootstrapping procedures with the aid of Hayes’ (2013) PROCESS. Bootstrap results (see Table 3) indicate that, customer mistreatment’s indirect effect on family satisfaction via WFC was significant (point estimate = −0.09, 95% CI [−0.16, –0.04]). The Sobel test also reinforced this significant indirect effect (Sobel *Z* = −3.05, *p* < 0.01). Meanwhile, bootstrap results also demonstrated that, after controlling for WFC, the direct effects of customer mistreatment on family satisfaction were not significant (point estimate = −0.06, 95% CI [–0.19, 0.08]). Thus, WFC is regarded as a “full” mediator in the customer mistreatment-family satisfaction relationship. Hence, *H3* is supported.

#### Testing of Moderation and Moderated Mediation

For *H4*, we predicted that psychological detachment moderates the relationship between customer mistreatment and WFC. To test this hypothesis, we conducted a moderated multiple regression analysis. We centered all primary predictor variables before computing cross-product terms (Aiken and West, 1991). As summarized in Table 4, the addition of customer mistreatment × psychological detachment interaction in Step 2a of the analyses explained a significant amount of incremental variance in WFC (β=–0.23, *p*<0.01, Δ*R*^2^=0.04). For *H5*, Leader-member exchange was predicted to play a moderating role in the relationship between customer mistreatment and WFC. As seen in Table 4, the addition of customer mistreatment × LMX in Step 2b of the analyses explained a significant amount of incremental variance in WFC (β = −0.15, *p* < 0.01, Δ *R*^2^ = 0.02), indicating support for H5.

**TABLE 4:** Result of moderated regression analysis.

**Variable**	**Dependent variable: work-to-family conflict**			
	
	***Step 1a***	***Step 2a***	***Step 1b***	***Step 2b***
Gender	–0.03	–0.02	–0.02	–0.03
Age	0.02	0.03	0.02	0.02
Marital status	0.01	0.01	0.01	0.02
Customer Mistreatment	0.37^∗∗^	0.34^∗∗^	0.63^∗∗^	0.62^∗∗^
Psychological detachment	–0.42^∗∗^	0.44^∗∗^		
Customer Mistreatment ×		–0.23^∗∗^		
Psychological detachment				
Leader-member exchange			–0.63^∗∗^	0.59^∗∗^
Customer Mistreatment ×				–0.15^∗∗^
Leader-member exchange				
*R*^2^	0.31	0.35	0.44	0.46
Δ *R*^2^		0.04		0.02

**N* = 221; ^∗∗^*p* < 0.01.*

**FIGURE 2:**
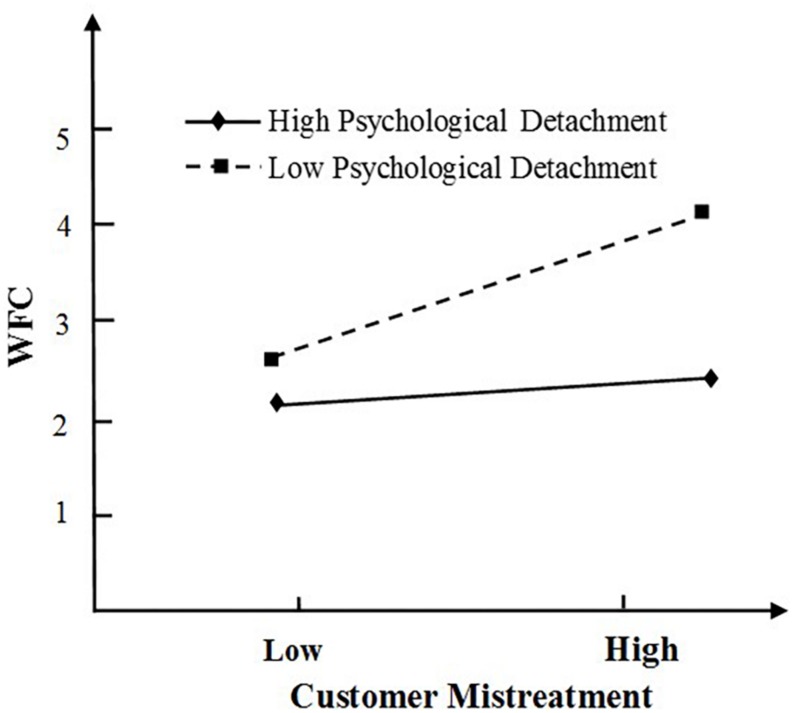
Interaction between customer mistreatment and psychological detachment on WFC.

**FIGURE 3:**
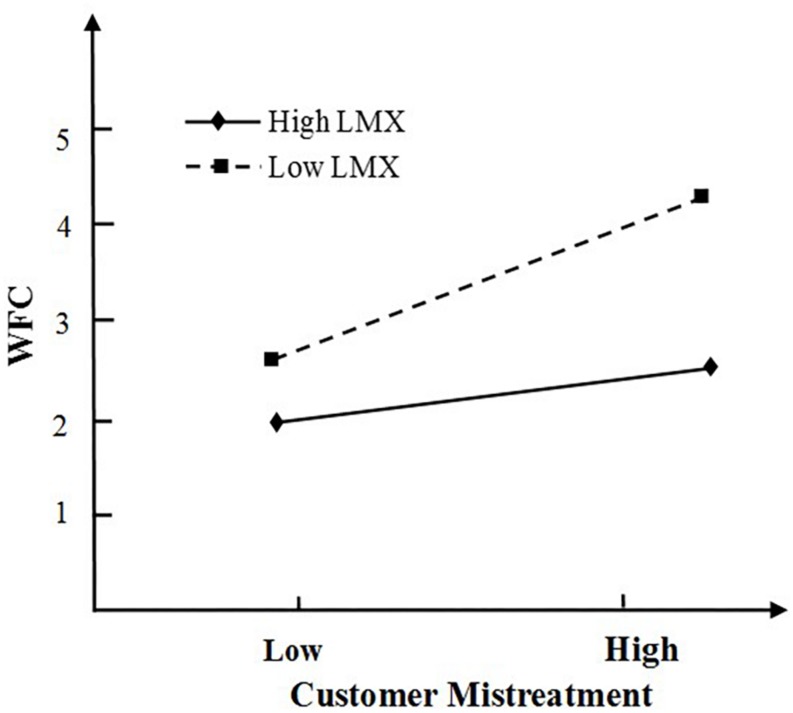
Interaction between customer mistreatment and LMX on WFC.

As proposed by Aiken and West (1991), we then plotted the moderation effect of psychological detachment and that of LMX in Figures 2, 3 respectively. As illustrated in Figure 2, mistreated employees with high levels of psychological detachment reported lower WFC levels than those with low levels of psychological detachment. Thus, H4 is supported. Similarly, as predicted, a graph of the interaction indicated that the relationship between customer mistreatment and WFC was weaker for individuals high in LMX than those low in LMX (see Figure 3). Thus, H5 is supported.

To validate the moderated mediation relationships (*H6* and *H7*), as proposed by Preacher et al. (2007), we examined the conditional indirect effects of customer mistreatment on family satisfaction via WFC at high and low values of psychological detachment and LMX respectively. Results (see Table 5) indicate that the indirect effects of customer mistreatment on family satisfaction via WFC decreased as psychological detachment increased from 2.31 (–1 *SD*; point estimate = –0.15; 95% CI [– 0.25, – 0.07]) to 4.05 (+1 *SD*; point estimate = –0.03; 95% CI [−0.09, 0.01]). At high level of psychological detachment, the indirect effects of customer mistreatment on family satisfaction were no longer statistically significant. Therefore, Hypothesis 6 is supported. Likewise, Table 5 indicate that the indirect effects of customer mistreatment on employees’ family satisfaction via WFC decreased as LMX increased from 2.48 (−1 *SD*; point estimate = −0.20; 95% CI [−0.33, −0.09]) to 3.92 (+1 *SD*; point estimate = −0.12; 95% CI [−0.22, −0.06]). Therefore, Hypothesis 7 is supported.

**TABLE 5:** Conditional indirect effects at high and low levels of psychological detachment and leader–member exchange for customer mistreatment.

**Moderator**	**Level**	**Conditional indirect effect**	**Boot SE**	**95% CI**	
				
				**LL**	**UL**
Psychological detachment	Low (mean − 1 *SD*)	−0.15^∗∗^	0.04	−0.25	−0.07
	High (mean + 1 *SD*)	−0.03	0.02	−0.09	0.01
Leader-member exchange	Low (mean −1 *SD*)	−0.20^∗∗^	0.06	−0.33	−0.09
	High (mean + 1 *SD*)	−0.12^∗∗^	0.04	−0.22	−0.06

**N* = 221; ^∗∗^*p* < 0.01; *SD*, standard deviation; CI, confidence interval; LL, lower limit; UL, upper limit. Bootstrap sample size = 1000.*

## Discussion and Conclusion

The aim of our study was to bridge research gaps concerning the work-family effects of customer mistreatment among service employees. We developed an integrated model that explored both the mechanism linking customer mistreatment to family satisfaction and the moderating effects of psychological detachment and LMX. By applying the S-S-O framework, as expected, the study findings reveal that WFC would act as a mediator between customer mistreatment and family satisfaction, and that psychological detachment and LMX would attenuate both the direct effects of customer mistreatment on WFC and the indirect effects of customer mistreatment on family satisfaction via WFC.

### Theoretical Implications

First, we believe that our study has contributed to the evolution of the customer mistreatment construct by providing evidence of its detrimental effects on family domain for the organization’s members. Previous research has mainly tended to focus on customer mistreatment’s harmful effects to the employees’ work domain (e.g., Grandey et al., 2005; Shao and Skarlicki, 2014). We extend this line of research by showing that customer mistreatment can spill over into the home arena for front-line workers in the service sector, which supported the work-family “spill-over” model (e.g., Wu et al., 2012; Liu et al., 2013). More specifically, for service employees, customer mistreatment may cause a paucity of resources by a WFC increase, and then this leads to the decrease of employees’ family satisfaction. The current study provides first longitudinal evidence that customer mistreatment has detrimental effects on employees’ family domain (e.g., family satisfaction).

Second, the research provides direct insight into WFC mediation in the customer mistreatment-family satisfaction relationship. Consistent with the work-family interface model (Parasuraman et al., 2010), WFC is a psychological mechanism linking customer mistreatment and family satisfaction. As indicated in our study, if one is mistreated when interacting with customers, this mistreatment may result in a WFC increase, which in turn decreases one’s perceived feeling of family satisfaction. In addition, the current study applies the S-S-O framework to the argument. The finding verifies the relationships of customer mistreatment as job stressor, WFC as job strain, and family satisfaction as job outcome, demonstrating that the S-S-O framework can be a holistic approach to understand the intervening mechanism between work-domain stressors and home-domain outcomes among Chinese service employees.

Third, to answer the call by Shao and Skarlicki (2014) to explore if psychological detachment can help people to better cope with customer mistreatment, we examined psychological detachment moderating customer mistreatment’s effects on individuals (e.g., WFC). In agreement with Sonnentag et al. (2010)’s argument of psychological detachment as a protective factor in the stressor–strain relation, the current study findings indicate that psychological detachment at non-work time might protect employees against customer mistreatment. To the best of our knowledge, this study is the first to provide meaningful insights into the interaction between customer mistreatment and psychological detachment in predicting WFC and family satisfaction, and suggests that job stressors (e.g., customer mistreatment) are less harmful when employees mentally disengage from their job during off-job time.

Fourth, we extend past research by examining the moderating effect of LMX on employee reactions to customer mistreatment. Martin et al. (2010) have, early on, called for a more thorough exploration of the role of LMX as a moderator. Although there exists rich literature related to the LMX variable, no research to date has explored LMX’s buffering role in the relationship between customer-related work stressors (i.e., customer mistreatment) and family related outcomes (i.e., WFC and family satisfaction). Our study indicates that LMX is an important buffer against the adverse spill-over effects of customer mistreatment on service employees. Individuals with high LMX tend to receive more social resources (e.g., support and trust) from their supervisors than those with lower levels, and are thus less likely to react negatively to customer mistreatment. The results support the COR theory that social resources could facilitate the conserving of one’s resource reservoir and thus aid stress resistance (Hobfoll, 1989).

### Practical Implications

Results from the present study also have implications for managerial practices.

First, the organizations should recognize the potential harmful consequences of customer mistreatment on the targets’ family domain. Our research shows that for the service employees, WFC and lack of family satisfaction can arise from the unfair and unjust treatment by customers. Irrational and inconsolable customers always exist, and customer mistreatment is thus difficult to prevent (Park and Kim, 2019). However, the organizations should try to reduce the occurrence of customer mistreatment in case of its negative work-family spill-over effects for organizational employees. The organizations can institute a service policy informing customers that mistreating employees will not be tolerated (Shao and Skarlicki, 2014). For instance, a blacklist of the negatively behaving customers with punishments may deter customer mistreatment. What is more, this policy might denote a kind of social support that helps employees to cope with customer mistreatment. Meanwhile, organizations can carry out assessments of the customer mistreatment level among service employees, for it offers important diagnostic information to identify those undergoing severe mistreatment, and organizations could then work with them to better cope with the mistreatment. Besides, mitigating the subjective experiences of customer mistreatment may provide a solution if mistreating behaviors by customers could not be avoided or eliminated. One recent research by Song et al. (2018) suggested that employees’ subjective experience of customer mistreatment is distinct from actual customer mistreatment behaviors, and it can be reduced by the recall of prosocial action and taking the perspective of customers. As such, employees are encouraged to be mindful of the needs of customers and “step into the shoes of” customers through education and training schemes.

Second, our study highlights the importance of enabling service employees to disengage from job-related thoughts during off-job time. As indicated in the study, individuals who psychologically detach more frequently are less likely to react to customer mistreatment by an increase of WFC, and to experience lower family satisfaction. Improving employees’ psychological detachment during off-job time will largely fall on management shoulders. The organizations should provide training to develop employees’ skills in ‘cleansing’ their minds. To be engaged in off-job activities requires one’s full presence and awareness, which might increase psychological detachment from work (Sonnentag et al., 2010). To engage oneself in one task long enough to get absorbed may help distract one’s negative thoughts and break the cycle of rumination (Baranik et al., 2017). Toward this end, employees are encouraged to actively devote attention to a non-work-related task (e.g., participating in sport or engaging in volunteer work) or engaging in daily transition rituals (e.g., winding down at the end of the working day). The daily practices can also help one to psychologically detach from work and recover from stressful work experiences (Kuhnel and Sonnentag, 2011). As such, it may be beneficial for employees to cleanse their minds during break time in the workplace. Management can also establish non-work space within the organization (e.g., employee recreation facilities) that can help employees to be mentally disengaged with job stressors.

Third, considering the boundary conditions of LMX with respect to customer mistreatment, our research informs that organizations should endeavor to foster and facilitate a mutually respectful and supportive relationship between leaders and their followers. On the one hand, to facilitate the establishment of high-quality LMX relationships, organizations can hold social activities or gatherings (e.g., team travel, dining out, and New Year Parties) in which both leaders and followers are involved and through which mutual respect and trust between two parties can be promoted. Moreover, the quality of the leader-follower relationship might be imbedded into the performance assessment system of team building and supervisors. For instance, organizations can encourage the supervisors who have high LMX relationship with subordinates by material and spiritual rewards (e.g., rating them as “excellent mentor/supporter”). On the other hand, supervisors themselves should foster close and positive relationships with their subordinates. In practice, supervisors need to communicate regularly with their subordinates, show concern for subordinates’ needs, and assist subordinates in solving work-related problems.

### Limitations and Future Research

As is the case with any study, this one is also not without its limitations. First, all variables in our model are self-reported, and thus the CMV could be a concern. However, in the study the time lagged design (i.e., three points in time for data collection) was employed to reduce CMV problem (Podsakoff et al., 2003). In addition, according to Schaubroeck and Jones (2000), CMV is unlikely to explain interaction effects, which are the main focus of this study. Thus, we believe CMV did not raise a major concern for this study. The study is focused on employee perceived feeling of customer mistreatment, WFC, and family satisfaction, and thus the self-report measurement may be the most valid for the model variables. However, future research might consider the intensive longitudinal methods (e.g., experience sampling methodology or diary study) for the self-report data collection. As a valuable addition to more traditional self-report measures (e.g., retrospective self-reporting method), the intensive longitudinal methods involve sequences of repeated measures and may help unpack the influences of customer mistreatment on employees’ actual experiences.

Second, our study sample are front-line employees in the beauty and hairdressing industry and they exclusively have face-to-face interaction with customers, which represents a potential limitation. Customers and service employees can behave differently in telephone versus face-to-face interactions (Harris and Reynolds, 2003). In comparison to voice-to-voice encounters, face-to-face encounters are more complex and difficult for service employees (Diefendorff et al., 2019). For instance, Grandey et al. (2004) argued that voice-to-voice employee-customer interaction might minimize the possibility that one is physically harmed by an aggressive customer. Thus, we need to further examine the generalizability of our findings to the sectors of verbal interaction with customers (e.g., call centers).

Third, though the time-lagged research design is applied in the current study, the causal relationships between the key constructs should be interpreted with caution. The undesirable family related experiences may also have spill-over effects on the work domain. For example, employees experiencing WFC may decrease their devotion to the job (e.g., lowering the customer service quality), thereby engendering the mistreatment by customers. Thus, to understand the causality among the key constructs, researchers are encouraged to undertake longitudinal studies measuring all key variables across time in the future.

Despite these limitations, our study is informative in that it identified how customer mistreatment exerts a detrimental influence on the service employees’ family domain. According to our literature review, this study represents the first known empirical study examining the roles of psychological detachment and LMX in buffering customer mistreatment work-family spill-over effects on individuals. Additionally, as exposure to customer mistreatment has negative implications for the targets’ family lives, organizations should make efforts to reduce and help employees cope with customer mistreatment if its occurrence is unavoidable.

## Author Contributions

RZ was responsible for the designing and writing. YW was responsible for the literature review, data collecting and analysis. KF-M was responsible for English language editing.

## Conflict of Interest Statement

The authors declare that the research was conducted in the absence of any commercial or financial relationships that could be construed as a potential conflict of interest.
